# Precise Orbit Determination of MEX Flyby Phobos Using Simulated Radiometric and Image Data

**DOI:** 10.3390/s21020385

**Published:** 2021-01-08

**Authors:** Xinbo Zhu, Lu Liu, Suyan Liu, Pan Xie, Wutong Gao, Jianguo Yan

**Affiliations:** 1 The college of astronautics, Nanjing University of Aeronautics and Astronautics, Nanjing 210016, China; xinberg@163.com; 2 Shanghai Institute of Satellite Engineer, Shanghai 201100, China; wakexie@163.com; 3 State Key Laboratory of Information Engineering in Surveying, Mapping and Remote Sensing, Wuhan University, Wuhan 430070, China; yls0712@163.com (S.L.); gaowutong@whu.edu.cn (W.G.); jgyan@whu.edu.cn (J.Y.)

**Keywords:** mars express spacecraft, flyby, image data, precise orbit determination

## Abstract

A navigation camera or topography camera is a standard payload for deep space missions and the image data are normally used for auto-navigation. In this work, we study the potential contribution of image data in precise orbit determination for deep space spacecraft. The Mars Express (MEX) spacecraft has generated extensive Phobos image data during flybys of Phobos, but these data have not been used in precise orbit determination because of the difficulty in employing these image data. Therefore, we did an experiment using simulated image data as the first step for exploring how to use real image data in precise orbit determination of spacecraft. Our results demonstrate that image data can provide stronger constraints on orbit in the tangential and normal directions than Doppler data. When the image data were used in the MEX orbit determination during the MEX Phobos flyby, the orbit determination accuracies in the tangential and normal directions were significantly improved. This work will provide a reference for real image data processing during MEX Phobos flyby to improve MEX orbit accuracy as well as Phobos ephemeris accuracy.

## 1. Introduction

Mars is the planet nearest to the Earth and the focus of many deep space exploration missions. Since the 1970s, Phobos, the natural satellite closest to Mars has been receiving more and more attention from researchers [1,2,3,4,5,6,7,8]. The Mariner 9 mission in 1972 took a close-up shot of Phobos for the first time [1]. NASA launched the Viking 1 and Viking 2 in 1975, and a large number of Phobos images were obtained during the Viking missions. These data were used to construct an original Phobos shape model [2]. Russia launched the Phobos 1 and Phobos 2 missions in 1988, but only a few images of Phobos were collected by Phobos 2 [3]. The Mars global surveyor (MGS) launched in 1996 collected many images using an onboard camera as it entered the orbit of Mars [4]. The Mars Express (MEX) mission launched in 2003 is the first Mars probe launched by the European Space Agency (ESA). The MEX spacecraft will fly close to Phobos every five months [5] and is equipped with a high-resolution stereo camera that can provide images for precision shape modeling of Phobos [6]. The Mars Reconnaissance Orbiter (MRO) was launched in 2005 and multiple high-resolution images of Phobos were collected using high-resolution cameras (HiRISE) [7]. 

Precise orbit determination of the spacecraft in deep space exploration missions is essential for the success of the mission as well as scientific interpretation of remote sensing data [9]. Precise orbit determination is typically carried out by using radiometric Doppler and range measurements from Earth tracking stations, but there are some shortcomings. In the conventional two-way or three-way measurement mode, as the Earth-Sun-spacecraft angle approaches 180°, the spacecraft is blocked by the Sun, making it impossible for ground stations to receive these measurements. Moreover, as the Sun-Earth-spacecraft angle approaches 0°, the radio tracking data of the spacecraft will be significantly influenced by solar plasma, so the accuracy of orbit determination is reduced when these tracking data are used in orbit determination of spacecraft [10,11]. Radiometric data are also subject to the scheduling availability of tracking stations that typically support different missions simultaneously. Compared with radiometric data, image data from the high-resolution airborne camera on-board the spacecraft has the advantage of not being affected by propagation media. Its accuracy relates to flying altitude, camera parameter accuracy, and camera attitude accuracy. When image data from the high-resolution airborne camera on-board the spacecraft is of sufficient quality, it can be used as an additional orbit constraint to compensate for the shortcomings of radiometric data from ground tracking stations for precise orbit determination. 

Images from spacecraft have been used to improve orbit knowledge on many deep space exploration missions [10,11,12,13,14,15,16,17,18,19]. In Near Earth Asteroid Rendezvous (NEAR) and Dawn missions, the gravity field and mass of the target body, the orbit of the target body around the Sun, the orientation and spin of the target, the location of the optical landmarks on the surface of the target, and orbits of spacecraft were jointly determined using earth-based tracking and optical landmark tracking data in a global estimation procedure [11,12,13]. In their work, the optical landmark tracking data made a significant contribution to the parameter solutions. In support of the Origins, Spectral Interpretation, Resource Identification, Security, Regolith Explorer (OSIRIS-Rex) mission, the spacecraft orbit and Bennu ephemeris was determined using radiometric and optical measurements [14]. Subsequently, the Bennu ephemeris was refined using synthetic astrometric observations, pseudo-range points, and a ground-based observational dataset [15]. This shows that optical measurements not only make a contribution in orbit determination of spacecraft but also contributed to refinements in the Bennu ephemeris. The precise orbit of the Hayabusa2 spacecraft with respect to asteroid Ryugu was dynamically determined using the data sets collected by the laser altimeter (light detection and ranging, LIDAR) onboard the spacecraft and automated image tracking (AIT). The results show all six components of the initial state vector can be derived stably using LIDAR and AIT data, which is difficult to achieve using only LIDAR data or AIT data [16]. Currently, there is no research addressing the use of Phobos image data in precision orbit determination, although the MEX mission has provided a large amount of image data for this target. To explore how to use real image data in precise orbit determination of the MEX during the MEX Phobos flyby, we did an experiment using simulated image data as the first stage of an ongoing project.

In the paper, we present a method for precise orbit determination using image data from MEX spacecraft during the flyby of Phobos. We compared orbit determination results from the combination of Doppler data and image data and results from methods using Doppler data. The rest of the paper is organized as follows: the process of image data simulation and the methodology of image orbit determination are introduced in Section 2. Comparative results and discussion are in Section 3 and the conclusions are drawn in Section 4.

## 2. Data and Methodology

### 2.1. Basic Data for Simulation

To employ the image data in precise orbit determination, we need to simulate image feature points and the corresponding surface feature points. Three kinds of basic data are required to simulate these two kinds of data: a Phobos shape model, MEX flyby orbits, and the geometric properties of the super resolution channel (SRC) camera.

#### 2.1.1. Phobos Shape Model

The Phobos shape model in this work is a spherical harmonic function model up to degree and order 45 as adapted from Willner et al. [20]. We computed 16,022 coordinate points on the surface of the model in a grid 2° by the analytic expression [7]. The reference coordinate system of this Phobos shape model is the Phobos-fixed coordinate system, as defined by the International Astronomical Union (IAU). The +X-axis is oriented to Mars, and the Z-axis is coincident with the mean revolution axis. Longitude is measured westward. As it is a right-handed coordinate system, the direction of the Y-axis is fixed. 

#### 2.1.2. MEX Flyby Orbits

The MEX spacecraft carried out a flyby of Phobos on 29 December 2013. The nearest distance to Phobos during the flyby was 59 km. The MEX ephemeris for the MEX Phobos flyby was generated through orbit integration with a 5 s integration step, based on an initial MEX state and highly precise force models, as shown in Table 1.

The initial MEX state refers to Cartesian coordinates and velocities of MEX in Mars J2000 at reference epoch. The force models include the latest JPL Martian gravity field model [21], the third-body perturbations from the Sun, and the large planets and large asteroids [22]. The post-Newtonian effect [23], the Mars solid tide perturbation [21], and the atmospheric drag [24] were also considered. The solar radiation pressure and thermal Martian albedo as well as indirect radiation (IR) were modeled as in Montenbruck & Gill [25] and also included in the orbit integration. 

The pixel scale of images varies with the spacecraft′s altitude and velocity with respect to the planetary body. The spacecraft′s altitude during the MEX Phobos flyby could change greatly over a short time span. For image simulation, we selected the orbital data from 2013-12-29 07:07:35–07:10:25 to avoid low image resolution caused by high spacecraft altitude. Table 2 summarizes the information related to the MEX flyby orbit used in the image simulation. 

The information includes the period of arc, sample interval of orbit, number of orbit points, and spacecraft altitude.

#### 2.1.3. Geometric Properties of the SRC Camera

The geometric properties of the SRC camera used in the simulation are shown in Table 3. 

The SRC has a nominal focal length of 975.0 mm. An in-flight recalibration of the focal length indicated a significantly larger focal length of 988.5 mm. The image focal point is defined by the geometric center of 1024 by 1024 pixel sized CCD array: $x_{0}=y_{0}=512.5$. The edge pixels of the CCD array were used to measure dark current leaving an area of 1008 lines with 1018 samples each, defining the active image area of the CCD. One pixel of the SRC had a field of view of 9 µrad correspondings to a field of view of 0.5° for the entire image.

### 2.2. Image Feature Point Simulation

We did not process the real images to obtain the image feature points but directly simulated the image feature points based on the geometric properties of the SRC camera. Since the orbital data was limited during the flyby, there are few images. We assumed that there are 150 randomly distributed image feature points in each image to increase the number of image feature points. For the 35 orbit points simulated for the 2013 flyby, 5250 image feature points were generated. Noise was also added to approximate real image feature points. The noise sources added in the simulation of image feature points and their values are given in Table 4. 

As shown in Table 4, three kinds of noise items were considered in the simulation, image noise, Phobos shape error, and camera attitude errors.

### 2.3. Simulation of the Surface Feature Point

This paper is based on the imaging model to simulate the surface feature points. In the following subsections, we describe the steps in detail. 

#### 2.3.1. Unify Coordinate System

**Figure 1:**
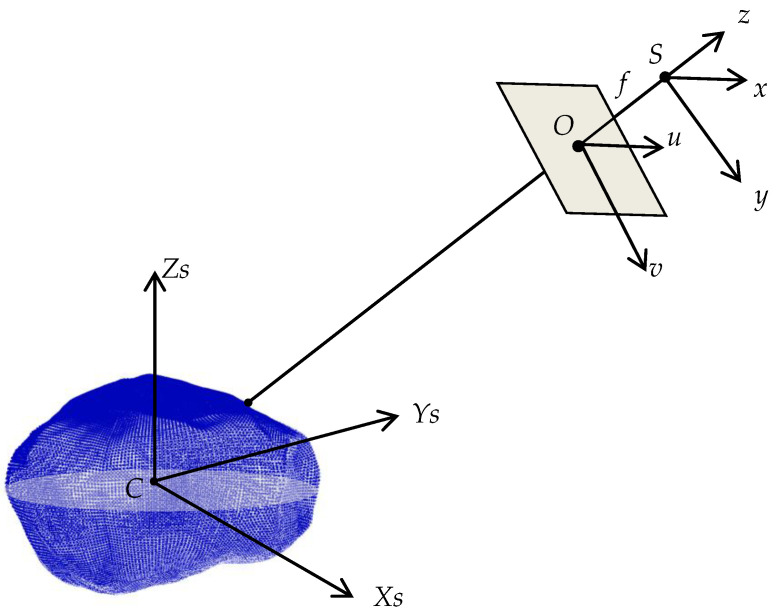
Schematic diagram of the Phobos imaging model. The *S-xyz* is the image space coordinate system that uses the projective center (*S*) as its origin. The *O-uv* is the photo coordinate system and the origin of it is the principal point (*O*) of the image. The *C-XsYsZs* is the Phobos-fixed coordinate system defined by IAU. The f is the focal length of the camera. The gray plane is the image plane. The blue ellipsoid is the shape model of Phobos.

Image generation simulates a process that captures a static picture instantly in time and space; thus, the simulation of the surface feature point corresponding to the image feature point needs to be completed in the Phobos-fixed coordinate system. The imaging model of Phobos is shown in Figure 1.

The imaging model involves three coordinate systems (as shown in Figure 1): the image space, the photo, and the Phobos-fixed coordinate systems. The image coordinates in the photo coordinate system had to be transformed into a Phobos-fixed coordinate system. Since image coordinates (*x*, *y*) in the photo coordinate system always correspond to image coordinates (*x, y*, *−f*) in the image space coordinate system, the image space coordinate was used as a transition coordinate system to help the transformation from photo coordinate system to the Phobos-fixed coordinate system. The image space coordinate system can be transformed into the Phobos-fixed coordinate system using Equation (1):

(1) $$\left[\begin{array}{l} X\\ Y\\ Z \end{array}\right]=R_{r}\times \left(\begin{array}{l} x\\ y\\ -f \end{array}\right)+R_{0}$$

where *X*, *Y*, and *Z* are image coordinates in Phobos-fixed system and *x*, *y*, and *z* are image coordinates in image space coordinate system. The $R_{r}$ is the rotation parameter matrix and $R_{0}$ is the displacement parameter matrix. $R_{r}$ and $R_{0}$ were derived as follows: Assume the coordinates of the projective center are $\left(X_{S},Y_{S},Z_{S}\right)$ in the Phobos-fixed coordinate system. The coordinates of the projective center in the image space coordinate system are (0,0,0). Thus, the rotation parameter matrix $R_{r}$ and displacement parameter matrix $R_{0}$ can be derived as shown in Equations (2) and (3), given that the direction of the z-axis of the image space coordinate system is the direction from the origin of the Phobos fixed coordinate system to the projective center.

(2) $$R_{r}=\left[\begin{array}{l} r_{11}\;r_{21}\;r_{31}\\ r_{12}\;r_{22}\;r_{32}\\ r_{13}\;r_{23}\;r_{33} \end{array}\right]=\left[\begin{array}{ccc} \frac{Z_{s}}{\sqrt{X_{s}{}^{2}+Z_{s}{}^{2}}} & \frac{-X_{s}*Y_{s}}{\sqrt{X_{s}{}^{2}+Z_{s}{}^{2}}*\sqrt{X_{s}{}^{2}+Y_{s}{}^{2}+Z_{s}{}^{2}}} & \frac{X_{s}}{\sqrt{X_{s}{}^{2}+Y_{s}{}^{2}+Z_{s}{}^{2}}}\\ 0 & \frac{\sqrt{X_{s}{}^{2}+Z_{s}{}^{2}}}{\sqrt{X_{s}{}^{2}+Y_{s}{}^{2}+Z_{s}{}^{2}}} & \frac{Y_{s}}{\sqrt{X_{s}{}^{2}+Y_{s}{}^{2}+Z_{s}{}^{2}}}\\ \frac{-X_{s}}{\sqrt{X_{s}{}^{2}+Z_{s}{}^{2}}} & \frac{-Y_{s}*Z_{s}}{\sqrt{X_{s}{}^{2}+Z_{s}{}^{2}}*\sqrt{X_{s}{}^{2}+Y_{s}{}^{2}+Z_{s}{}^{2}}} & \frac{Z_{s}}{\sqrt{X_{s}{}^{2}+Y_{s}{}^{2}+Z_{s}{}^{2}}} \end{array}\right]$$

(3) $$R_{0}=\left[\begin{array}{c} X_{S}\\ Y_{S}\\ Z_{S} \end{array}\right]$$

where *r*_ij_ (i = 1, 2, 3; j = 1, 2, 3) are nine components corresponding to the rotation matrix. Moreover, the orbit coordinates of MEX are in the Mars J2000 coordinate system. This orbit will be transformed into the Phobos-fixed coordinate system, following IAU2015 [27].

#### 2.3.2. Surface Feature Point Interpolation

**Figure 2:**
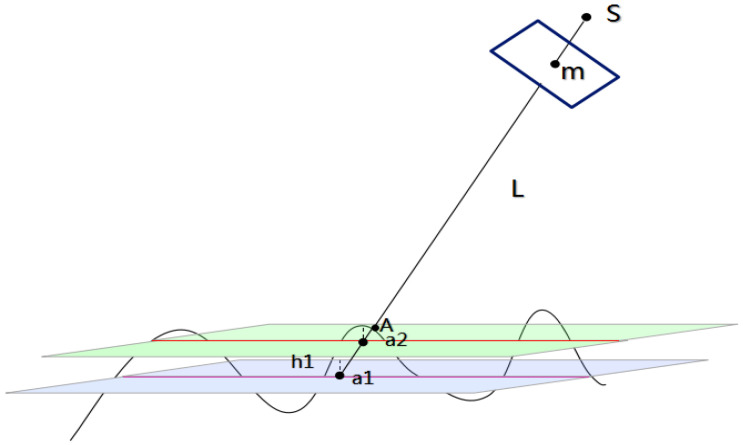
Schematic diagram of surface feature point interpolation. The **S** is the projective center. The **m** is an image feature point in the image. The **A** is a surface feature point corresponding to image feature point **m**. The **L** is a line connecting an image feature point **m** and the corresponding surface feature point **A**. The **a1** is the intersection of the line **L** and the mean elevation surface (shown in blue). The **a2** is the intersection of the line **L** and a new elevation surface (shown in green). The h1 is the elevation of point **a1**. The wiggly black line is a real elevation surface.

After unifying the coordinate system, all calculations were performed in the Phobos-fixed coordinate system. Figure 2 gives a diagram for interpolated surface feature points. 

The projection center **S**, an image feature point **m,** and the corresponding Phobos surface feature point **A** are collinear at the moment of photography as shown in Figure 2; thus, the steps for obtaining the surface feature point A are as follows. The line **L** connecting an image feature point **m** and the corresponding surface feature point **A** is constructed according to coordinates of projective center **S** and coordinates of image feature point **m**. The point sets of the local region including feature point **A** are obtained by intersecting line **L** with the Phobos approximate sphere model (radius R = 11,100 m). We calculate the mean elevation surface (*h_mean_*) for point sets of the local region, which intersects the line **L** at point **a1**; and the elevation *h*_1_ of point **a1** can be obtained by interpolating from the shape model. The *h*_1_ is set as the new elevation of the surface to calculate the difference of two elevation surfaces $\Delta h=h_{1}-h_{mean}$ and to get the intersection **a2** with the line **L**. If the difference between the new elevation surface and the previous elevation surface is less than 0.1 m, the process is stopped; otherwise, the process is repeated based on the new elevation obtained from **a2…ai**. The final intersection point is the surface feature point.

### 2.4. Image Observation Model

We constructed the image observation model and the corresponding partial derivatives to incorporate image data into the orbit determination filter. The method of constructing the image observation model is as follows: Suppose there are m image feature points in each image and the coordinates of the n-th image feature point in the i-th image are (*x*, *y*). We know that the projective center $\left(X_{S},Y_{S},Z_{S}\right)$, the image feature point (*x*, *y*), as well as the surface feature point $\left(X_{A},Y_{A},Z_{A}\right)$ are collinear. Therefore, the geometric relationship between the image feature point and the Phobos surface feature point can be constructed based on the collinearity. The image observation model [28] is obtained as follows:

(4) $$\begin{array}{l} x=-f\frac{r_{11}(X_{A}-X_{S})+r_{12}(Y_{A}-Y_{S})+r_{13}(Z_{A}-Z_{S})}{r_{31}(X_{A}-X_{S})+r_{32}(Y_{A}-Y_{S})+r_{33}(Z_{A}-Z_{S})}\\ y=-f\frac{r_{21}(X_{A}-X_{S})+r_{22}(Y_{A}-Y_{S})+r_{23}(Z_{A}-Z_{S})}{r_{31}(X_{A}-X_{S})+r_{32}(Y_{A}-Y_{S})+r_{33}(Z_{A}-Z_{S})} \end{array}$$

where *f* is the focal length of the camera. *r*_ij_ (i = 1, 2, 3; j = 1, 2, 3) are nine components corresponding to the inverse matrix of rotation matrix $R_{r}$. Assume that the position of the spacecraft is consistent with the coordinates of the projective center. From the image observation model, we know that when the position of the spacecraft, the focal length of the camera, and coordinates of surface feature point are known, then the surface feature point can be projected to the focal plane so that coordinates (*x*, *y*) in the photo coordinate system can be attained. Thus, the pixel coordinates (*r*, *c*) of the image feature point can be computed based on the pixel scale parameters of the camera.

### 2.5. Partial Derivatives of the Image Observation Model

Partial derivatives of the image observation model are computed as follows: Suppose the measured image feature point (x′, y′) at time *t* is **Obs**, then the corresponding image observation model (4) can be expressed simply as:

(5) $$Obs=G(X_{S},t)+\varepsilon$$

where G is computation value corresponding to **Obs** and is also the right part of Equation (4), which is a nonlinear function of $X_{S}$ and *t*; $X_{S}$ is state vector of MEX at time *t*, and ε corresponds to **Obs**. 

Due to the complexity of the model, it is hardly possible to directly solve any of these parameters from a given set of observations. It is therefore customary to linearize the relation between the observables and the independent parameters to obtain simplified expressions that can be handled more easily. We executed a Taylor expansion of Equation (5) at the referenced MEX state vector $X_{S}^{*}$ at time *t.* Thus, Equation (6) will be obtained by linearization with ignorance of the higher-order terms:

(6) $$Obs=G(X_{S}^{*},t)+H\cdot \varphi (t,t_{0})+\varepsilon$$

where **H** is a partial matrix of the calculation value with respect to the MEX state vector $X_{S}$ and $\varphi \left(t,\;t_{0}\right)$ is the state transition matrix. **H** and $\varphi \left(t,\;t_{0}\right)$ can be computed by Equations (7) and (8), respectively.

(7) $$\begin{array}{c} H={\frac{\partial G}{\partial X_{S}}|}_{X_{S}^{*}}=\\ {}[\begin{array}{cc} \frac{(Z_{A}a_{3}+(2X_{S}^{*}-X_{A})a_{4})a_{1}{}^{2}a_{2}{}^{2}f+(a_{2}{}^{2}-a_{1}{}^{2})a_{3}a_{4}X_{S}^{*}f}{a_{1}a_{2}{}^{3}a_{3}{}^{2}} & \frac{(2Y_{S}^{*}-Y_{A})a_{1}{}^{2}a_{4}f+a_{3}{}^{2}Y_{S}^{*}f}{a_{1}a_{2}a_{3}{}^{2}}\\ \frac{(2X_{S}^{*}-X_{A})(a_{2}{}^{2}a_{5}-a_{2}{}^{4}Y_{A}f)+(X_{A}Y_{S}^{*}-Y_{A}X_{S}^{*})a_{2}{}^{2}a_{3}f-a_{3}a_{5}X_{S}^{*}}{a_{2}{}^{3}a_{3}{}^{2}} & \frac{\left(X_{A}X_{S}^{*}+Z_{A}Z_{S}^{*})a_{3}f+(2Y_{S}^{*}-Y_{A})(a_{5}-a_{2}{}^{2}Y_{S}^{*}f\right)}{a_{2}a_{3}{}^{2}} \end{array}\\ \begin{array}{cc} \frac{((2Z_{S}^{*}-Z_{A})a_{4}-X_{A}a_{3})a_{1}{}^{2}a_{2}{}^{2}f+(a_{2}{}^{2}-a_{1}{}^{2})a_{3}a_{4}Z_{S}^{*}f}{a_{1}a_{2}{}^{3}a_{3}{}^{2}} & 0_{\left(1\times 3\right)}\\ \frac{(2Z_{S}^{*}-Z_{A})(a_{2}{}^{2}a_{5}-a_{2}{}^{4}Y_{A}f)-a_{3}a_{5}Z_{S}^{*}+(Z_{A}Y_{S}^{*}-Y_{A}Z_{S}^{*})a_{2}{}^{2}a_{3}f}{a_{2}{}^{3}a_{3}{}^{2}} & 0_{\left(1\times 3\right)} \end{array}] \end{array}$$

(8) $$\varphi (t,t_{0})=\frac{\partial X_{S}}{\partial X_{0}(t_{0})}$$

where $a_{1}=\sqrt{X_{S}^{*}{}^{2}+Y_{S}^{*}{}^{2}+Z_{S}^{*}{}^{2}}$, $a_{2}=\sqrt{X_{S}^{*}{}^{2}+Z_{S}^{*}{}^{2}}$, $a_{3}=X_{A}X_{S}^{*}+Y_{A}Y_{S}^{*}+Z_{A}Z_{S}^{*}-X_{S}^{*}{}^{2}-Y_{S}^{*}{}^{2}-Z_{S}^{*}{}^{2}$, $a_{4}=Z_{A}X_{S}^{*}-X_{A}Z_{S}^{*}$ and $a_{5}=fX_{A}X_{S}^{*}Y_{S}^{*}+fZ_{A}Y_{S}^{*}Z_{S}^{*}$. $X_{0}(t_{0})$ is the state vector of MEX at time $t_{0}$.

### 2.6. Simulation of Doppler Data

In our comparative experiment, we simulated two-way Doppler data received by the 70-m antenna of the NASA Deep Space Network (DSN) in Madrid (Spain) when the MEX was flying by Phobos. The two-way Doppler data were simulated based on the same highly precise force models (see Table 1) and precise knowledge of the ground station coordinates, tidal displacement [29], and the state-of-the-art tropospheric correction model VMF1 [30]. The arc span was from 2013-12-29 03:40:00 to 2013-12-29 12:30:00. The observation noise of 1 mm/s also was added to Doppler data. The final 5285 Doppler data were obtained by setting an observation interval of 5 s.

## 3. Results and Discussion

**Figure 3:**
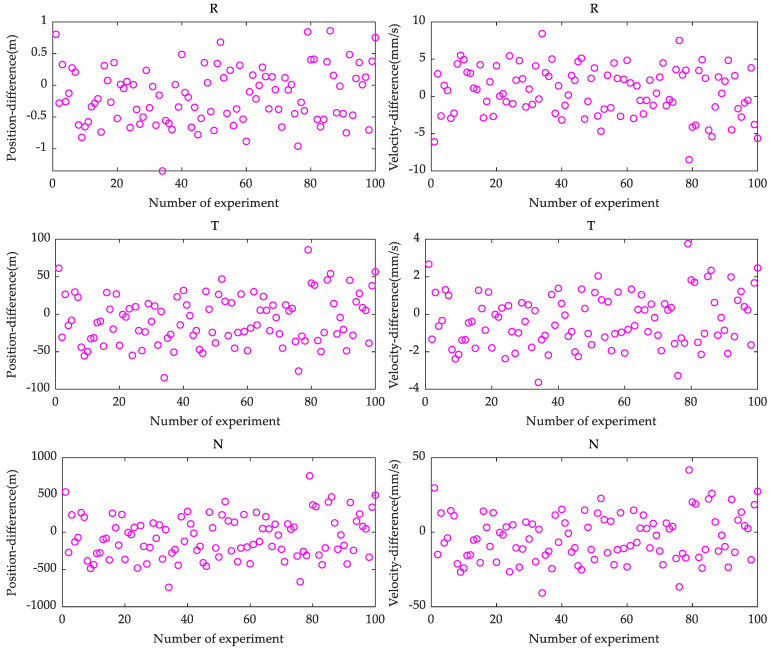
The orbital difference between initial spacecraft states computed with Doppler data and a “true” initial spacecraft state. “R”, “T”, and “N” denote radial, tangential, and normal directions, respectively. (Left: position difference, Right: velocity difference).

We evaluated the contribution of image data in MEX precise orbit determination based on the image data model as presented previously in Section 2.4. The MEX precise orbit determination using only Doppler and combination data (Doppler and image data) was carried out with the Mars spacecraft orbit determination and gravity field recovery system (MAGREAS) developed by Wuhan University [31]. MAGREAS uses an iterative weighted batch-least squares (WBLS) estimator to find an initial spacecraft state solution that results in the minimum residual variance for a given set of measurements. The performance of the MAGRREAS software was confirmed by cross-validation against the GEODYN II planetary spacecraft orbit determination and dynamic parameter solution platform, and it also has been employed to process MEX radio tracking data [31,32]. Moreover, a Monte Carlo method was applied to validate the capability of image data for MEX precise orbit determination during the MEX Phobos flyby [33]. We did 100 experiments for each strategy by adding 300 m and 0.1 m/s random noise to the initial position and velocity, respectively. A total of eight parameters were estimated; these included the initial spacecraft state, solar radiation scale factor, and Martian atmosphere drag scale factor. The orbit determination performance was evaluated by directly comparing initial spacecraft states computed from different data with a “true” initial spacecraft state in Table 1. Figure 3 shows orbital differences in the radial (R), tangential (T) and normal (N) directions between initial spacecraft states computed with only Doppler data and a “true” initial spacecraft state. 

As shown in Figure 3, the precise orbit determination results have the smallest variation amplitude of position difference in the radial direction and the smallest variation amplitude of velocity difference in the tangential direction if we only use Doppler data. The variation amplitude of position difference in the tangential and normal directions is one and two orders of magnitude larger than the variation amplitude of radial position difference, respectively. These indicate that Doppler data have stronger constraints on orbital position in the radial direction and orbital velocity in the tangential direction. During the flyby, the angle between the line-of-sight (LOS) and the MEX orbit plane was close to six degrees, which means an edge-on orbit, therefore, the high-precision orbital position in the radial direction can be estimated. With such an edge-on tracking geometry, the Doppler data is not sensitive to the normal direction; we can see the relatively big position and velocity difference in this direction in Figure 3, bottom. 

**Figure 4:**
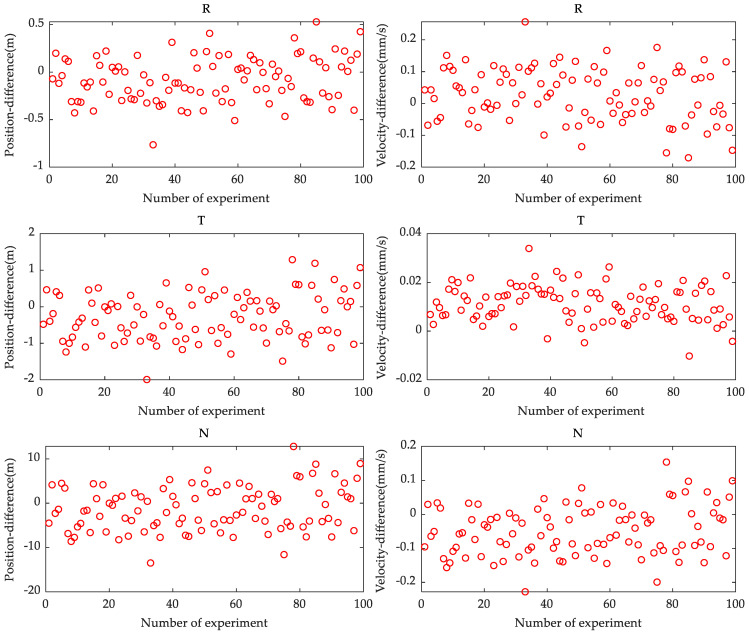
The orbital difference between initial spacecraft states computed with combined data (Doppler and image data) and a “true” initial spacecraft state. “R”, “T”, and “N” denote radial, tangential, and normal directions, respectively. (Left: position difference, Right: velocity difference).

Figure 4 shows orbital differences in the radial, tangential, and normal directions between the initial spacecraft states computed with the combined data and the “true” initial spacecraft state.

Figure 4 shows that the orbit determination results still have the smallest variation amplitude of position difference in the radial direction and the smallest variation amplitude of velocity difference in the tangential direction using the combination of Doppler and image data. However, comparing Figure 4 with Figure 3, we can see that there is an improvement in the orbit determination accuracy after including the image data. In particular, the orbital accuracies in tangential and normal directions were significantly improved. This indicates that the image data can provide stronger constraints on the orbit in the tangential and normal directions [19]. Additional analysis presented in this section will verify its contribution. 

We computed root mean square (RMS) of orbital position and velocity differences in the radial, tangential, and normal directions between the initial spacecraft states computed with different data and a “true” initial spacecraft state, as shown in Table 5. We can use Table 5 to quantitatively analyze the performance of precise orbit determination. 

From Table 5, unlike initial spacecraft states calculated using only Doppler data, the orbit determination results obtained by a combination of Doppler and image data yielded improved ratios of position accuracy in the three (radial, tangential, and normal) directions by 47.04%, 97.92%, and 98.26% respectively. Meanwhile, the improvement ratios for velocity accuracy in the three directions were 97.39%, 99.07%, and 99.46% respectively. Thus, the image data have a significant contribution to the orbital position accuracy in the tangential and normal directions, and the orbital velocity accuracy in all directions, while the contribution to the radial position accuracy is relatively small. This is because the orbit geometry can be optimized after including the image data. The Doppler data provides a stronger constraint in the radial direction because of the edge-on orbital geometry; whereas the image data constrain the orbit in the tangential and normal directions. The increase in the amount of data however, does not make the dominant contribution to the accuracy of orbit determination, as we did a test to extend the tracking arc by only using Doppler data to increase the amount of data. The results indicated that there was no significant improvement in orbit determination accuracy as compared to results that included image data.

**Figure 5:**
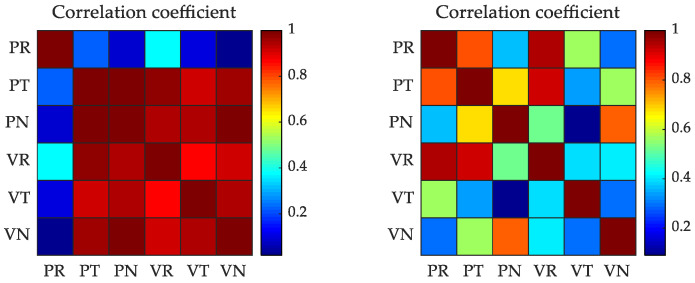
The correlation coefficient of the initial spacecraft states computed with different data (Left: Doppler data, Right: Doppler and image data). “R”, “T”, and “N” denote radial, tangential, and normal directions, respectively. (P: position, V: velocity).

To carry out a complete description of the results, the correlation of estimated parameters must be analyzed. Correlation coefficients of the initial spacecraft states computed with different data (only Doppler data, and Doppler data with image data) are presented in Figure 5.

From Figure 5, for the orbit determination results only using the Doppler data, there is a large correlation between other parameters, up to 0.9 or more, except for the radial position parameter. This shows that Doppler data is more sensitive to the estimation of radial position parameter, which is consistent with the results shown in Figure 3. For orbit determination using Doppler and image data, on the whole, the correlation between parameters was reduced. This result indicates that the image data improves the sensitivity of parameter estimation. These parameter correlation analysis results further lend support to the view that the MEX orbit determination accuracy in the MEX Phobos flyby can be improved after including the image data.

## 4. Conclusions

Precise orbit determination using Doppler data and image data for the 2013 MEX Phobos flyby was simulated based on the construction of the image observation model and our in-house planetary spacecraft precise orbit determination software MAGREAS. Precise orbit determination using conventional two-way Doppler data also was simulated for comparison. These results indicate that Doppler data place a stronger constraint on radial orbital position. There are significant improvements in orbital position accuracy in the tangential and normal directions after including the image data. Image data as a new orbit constraint is feasible, as demonstrated by a simulation of MEX precise orbit determination during the 2013 MEX Phobos flyby. 

This article is based on the results of simulation experiments, and the results show the reliability of our image data model using our in-house precise orbit determination software. In our future work, we will process real image data collected during MEX Phobos flyby and aim to improve the orbital accuracy of MEX as well as Phobos ephemeris. China’s Mars mission “Tianwen-1” is currently on the way to Mars; its orbit is similar to MEX. This spacecraft will conduct flybys of Phobos. Such simulation work will lay a solid foundation for processing our Mars mission data related to orbit determination for the “Tianwen-1” spacecraft and Phobos ephemeris refinement using the “Tianwen-1” flyby data.

## Figures and Tables

**Table 1:** The configuration of the orbit integration.

Configuration	Description
The initial MEX state (MARS J2000)	Epoch (UTC): 2013-12-29 03:40:00; X(m): 2067685.5850630, Y(m): −6081856.4673221, Z (m):10990534.6587460 Vx(m/s): −1085.32769224, Vy(m/s): −673.97767323, Vz(m/s): 490.54349005
Force model	MRO120D (truncated to 95 degrees and order); N-body perturbation (DE421, Phobos ephemeris: NOE-4-2015-b.bsp); Solar radiation (Simple model); Martian Albedo and IR; Post-Newtonian effect (Sun and Planets); Mars solid tidal perturbation (Love number K2 = 0.169); Mars atmospheric drag (atmospheric pressure and density from Mars Climate Data base v5.3)

**Table 2:** The information related to MEX flyby orbit.

Arc	The Flyby in 2013
Time span	2013-12-29-07:07:35-07:10:25
Sample interval	5 s
Number of orbit point	35
Spacecraft altitude	59–264 km

**Table 3:** Geometric properties of the SRC camera [26].

Property	Value
Focal length *f*	988.5 mm
	(in-flight calibration)
Number of pixels	1024 × 1024 pixels
Number of active pixels	1008 × 1018 pixels
	(lines × samples)
Pixel size	9 × 9 µm
FOV per pixel	9 µrad
FOV total	9 mrad

**Table 4:** Noise sources.

Item	Values
Image noise	0.5 pixel
Phobos shape error	1.0 m
Camera attitude errors	boresight: 1 pixel; twist angle: 1.0 mrad

**Table 5:** RMS of orbital difference between initial spacecraft states computed with different data types and a “true” initial spacecraft state. “R”, “T”, and “N” denote radial, tangential, and normal directions, respectively.

Data Type	Data Amount	Position (m)			Velocity (mm/s)		
		R	T	N	R	T	N
Doppler	5250	0.4715	33.5680	294.4105	3.3327	1.4512	16.2230
Doppler + Image	10,535	0.2497	0.6971	5.1336	0.0869	0.0135	0.0874

## Data Availability

The data presented in this study are available on request from the corresponding author.
